# Fibrillary Glomerulonephritis in Systemic Lupus Erythematosus: A Case Series

**DOI:** 10.1016/j.xkme.2025.101225

**Published:** 2025-12-16

**Authors:** Gashu Ayehu, Michelle Vanessa Aguirre Polo, Yihe Yang, Ming Wu, Kenar D. Jhaveri

**Affiliations:** 1 Glomerular Center at Northwell Health, Division of Kidney Diseases and Hypertension, Zucker School of Medicine at Hofstra/Northwell, Great Neck, NY; 2 Department of Nephrology, Hospital Universitario Fundación Jimenez Diaz, Madrid, Spain; 3 Department of Pathology, Weill Cornell Medical Center, New York, NY; 4 Department of Pathology, Northwell Health, NY

**Keywords:** Fibrillary glomerulonephritis, lupus nephritis, rituximab, SLE

## Abstract

Fibrillary glomerulonephritis (FGN) is a rare glomerular disease characterized by the deposition of nonamyloid, typically Congo red-negative fibrils within the glomerular basement membrane and mesangium. Although the pathogenesis of FGN remains incompletely understood, several autoimmune conditions have been associated with its development, including systemic lupus erythematosus (SLE). The co-occurrence of SLE and FGN is rare, and the underlying pathophysiologic link, if any, remains poorly understood. We report 2 cases of biopsy-proven FGN with positive DnaJ homolog subfamily B member 9 (DNAJB9) immunostaining in patients with established SLE, one with concurrent membranous lupus nephritis (LN) and the other without LN. Both patients were treated with rituximab, achieving complete proteinuria remission with preserved kidney function in one case and improved creatinine levels in the other. Three cases of SLE-associated FGN with positive DNAJB9 staining have been previously reported. Two others have been described as such, but without documentation of relevant clinical details such as DNAJB9 status, making them unsuitable for the current discussion. Our cases add to the literature supporting a potential association between SLE and FGN and represent the first published evidence of the safety and efficacy of rituximab in this rare condition.

Fibrillary glomerulonephritis (FGN) is characterized by the deposition of nonamyloid, typically Congo red-negative fibrils within the glomerular basement membrane and mesangium.^1^ Although frequently idiopathic, FGN has been associated with autoimmune conditions, hematologic malignancies, and infections.^2^, ^3^, ^4^ Systemic lupus erythematosus (SLE) represents one such association; however, this relationship remains poorly understood. Distinguishing FGN from lupus nephritis (LN) is clinically important because of distinct histopathologic features and differing therapeutic approaches.

## Case 1

A 67-year-old woman with SLE history presented with progressive kidney dysfunction. She had class III LN 30 years earlier, treated with prednisone and azathioprine, achieving sustained remission. She presented with increasing creatinine from 1.2 mg/dL to 2.5 mg/dL and urinary protein-creatinine ratio (uPCR) ∼0.7 g/g. Antinuclear antibodies, C3, C4, antineutrophil cytoplasmic antibodies, and double-stranded DNA (dsDNA) (16 IU/mL) were negative. She did not have extrarenal manifestations of SLE.

**Figure 1:**
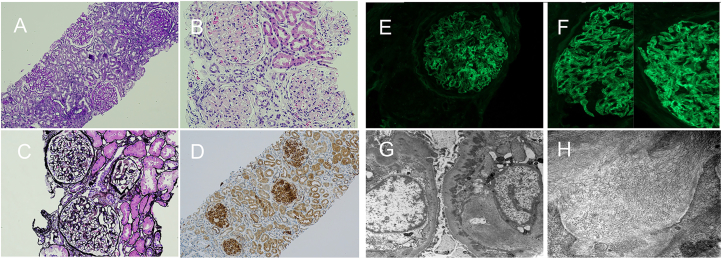
Kidney biopsy findings in 2 patients with systemic lupus erythematosus and fibrillary glomerulonephritis. Case 1: Slides A-D Case 2: Slides E-H (A) PAS stain showing glomeruli with mesangial matrix, cellularity expansion, mild interstitial fibrosis with PAS-positive hyaline casts and tubular atrophy (100×). (B) H&E stain showing glomeruli with mesangial matrix and cellularity expansion (200×). (C) Jones silver stain showing glomerular basement membranes with small holes without duplication, spikes, or double contours (400×). (D) Immunohistochemistry for DNAJB9 stain showing strong staining within glomeruli (100×). (E) Immunofluorescence staining for IgG shows smudgy mesangial and peripheral capillary loop positivity (4+). (F) Composite immunofluorescence panel showing polytypic light chain staining: kappa (left) and lambda (right). (G) Electron microscopy demonstrating fibrillary deposits in the mesangium and glomerular basement membranes. (H) Higher magnification electron microscopy shows nonbranching, randomly arranged fibrils measuring approximately 10-15 nm in diameter. Abbreviations: EM, electron microscopy; FGN, fibrillary glomerulonephritis; H&E, hematoxylin and eosin; IgG, immunoglobulin G; PAS, periodic acid–Schiff; SLE, systemic lupus erythematosus.

Kidney biopsy showed mesangioproliferative glomerulonephritis without endocapillary proliferation or crescents. Congo red staining was negative. Immunofluorescence showed smudgy IgG (4+), C3 (2+), kappa (4+), lambda (3+), and C1q (2+) staining in the mesangium and focally along capillary walls. Electron microscopy demonstrated minimal, segmental foot-process effacement. Mesangial and subepithelial electron-dense deposits of nonbranching, randomly arranged fibrils averaging 9.8 nm in diameter were seen. DNAJB9 staining was positive. Deposits were limited to glomeruli, with no immune-complex deposition in tubular or vascular compartments. Tubuloreticular inclusions were absent (Fig 1).

Treatment included supportive care with renin-angiotensin-aldosterone system blockade and rituximab (1 g intravenously ×2 doses). Proteinuria declined to uPCR 0.2-0.3 g/g and creatinine improved from 2.5 mg/dL to 1.6 mg/dL. Two additional maintenance rituximab doses over 2 years maintained uPCR <0.3 g/g, without complication over 31 months of follow-up.

## Case 2

A 55-year-old woman with SLE and class V LN who had had quiescent systemic disease for many years presented with mild and transient lupus rash, stable creatinine ∼ 0.5 mg/dL but increasing proteinuria, with a uPCR of 2.5 g/g. Additional evaluation showed an erythrocyte sedimentation rate of 75 mm/h, normal C3 and C4 levels, and negative dsDNA (<12 IU/mL).

Kidney biopsy showed early FGN and class V LN without endocapillary hypercellularity or crescents and with a National Institutes of Health lupus nephritis activity index of 0/24. Light microscopy showed thickened basement membranes with spike-like projections and mild mesangial expansion containing extracellular fibrillary deposits. Congo red staining was negative. Immunofluorescence showed finely granular IgG (4+) and C1q (1+) staining and minimal C3 staining. Electron microscopy demonstrated subepithelial and intramembranous electron-dense deposits with moderate foot-process effacement. Randomly arranged, nonbranching fibrils measuring 12-15 nm were seen in the mesangial and subendothelial spaces. Deposits were limited to glomeruli, with no immune-complex deposition in tubular or vascular compartments. DNAJB9 immunostaining was strongly positive.

She was treated with rituximab (1 g intravenously ×2 doses), in addition to continuing mycophenolate, hydroxychloroquine, renin-angiotensin-aldosterone system blockade, and blood pressure medications that she had been on for several months (she had declined steroids). Proteinuria gradually improved, with a uPCR of 0.2 g/g and creatinine remained stable within baseline range of 0.46-0.55 mg/dL. She remained in remission without the need for maintenance rituximab 34 months after her FGN diagnosis.

## Discussion

FGN is a rare but increasingly recognized glomerular disease, comprising 0.5%-1% of native kidney biopsies.^5^ It features randomly arranged, nonbranching fibrils (mean diameter 20 nm) that are typically Congo red negative, but rare Congo red-positive cases have been reported.^2^ Immunofluorescence shows polyclonal IgG and C3. Absence of Congo red staining and distinct fibril composition distinguish it from amyloidosis. Although historically idiopathic, FGN associates with diabetes (20%-24%), dysproteinemias (13%), autoimmune disorders (11%-15%), malignancies (9%-23%), and hepatitis C infection (3%-13%).^2^, ^3^, ^4^ Common autoimmune associations include SLE, Crohn disease, Graves’ disease, idiopathic thrombocytopenic purpura, and Sjögren syndrome.^4^^,^^6^, ^7^, ^8^, ^9^, ^10^ Although pathogenic mechanisms remain undefined, FGN represents an immune complex-mediated glomerulopathy with DNAJB9 as the key autoantigen. DNAJB9 presumably complexes with immunoglobulins and complement, initiating glomerular injury.^1^^,^^11^

In this case series, we present 2 patients with biopsy-proven FGN and a history of SLE without lupus nephritis (first case) and with concurrent membranous LN (second case). Of the 5 FGN cases diagnosed in our practice during the 3-year period and composing these cases, only the 2 reported here were associated with SLE. To date, only 3 such cases have been reported, all in female patients aged 17-53 years (mean 40 years) who presented with proteinuria ranging from 1.0-2.5 g/g and preserved or mildly impaired kidney function (Table 1).^12^, ^13^, ^14^ Notably, these cases demonstrated DNAJB9 positivity and fibril diameters of 15-20 nm, making the current report an important addition to this limited body of literature. It should be noted, however, that a few other cases have been described as such but were excluded from this review because of missing key details such as lupus serologies or DNAJB9 status.^15^^,^^16^

**Table 1:** Published Case Reports of Systemic Lupus Erythematosus-Associated Fibrillary Glomerulonephritis

Authors	Patient (Age/Sex)	Clinical Features	Serum Creatinine (mg/dL)	UPCR (g/g)	C3/C4	anti-dsDNA (IU/mL)	DNAJB9 IHC	Mean Fibril Diameter (nm)	Treatment	Outcome
Whelband et al^12^	50/F	Remote history of SLE; proteinuria	0.83	2.5	N/N	Negative	Positive	11.9-20	Steroids, mycophenolate	Stable kidney function; UPCR 0.5 g/g
Martin et al^13^	17/F	No prior history; malar rash, proteinuria	CrCl 246 mL/min^a^	2.4	↓/↓	243	Positive	18	RAASi, HCQ, steroids, mycophenolate	Stable kidney function; UPCR 0.65 g/g
Theodoropoulou et al^14^	53/F	No prior history; arthralgias, proteinuria	Normal	1.0	NR	Positive	Positive	11.8-18.8	Not reported	Not reported
Our case 1	67/F	SLE with prior LN; increasing SCr, proteinuria	2.5	0.7	↓/↓	16	Positive	9.8	RAASi, Rituximab	Improved kidney function; SCr 2.0 mg/dL; UPCR <0.3 g/g
Our case 2	55/F	SLE with prior LN; edema, proteinuria	0.47	2.5	N/N	<12	Positive	10-15	Rituximab, mycophenolate, HCQ, RAASi	Stable kidney function; SCr 0.46-0.55 mg/dL; UPCR 0.2 g/g

**Abbreviations:** anti-dsDNA, anti–double-stranded DNA antibody; CrCl, creatinine clearance; DNAJB9, DnaJ homolog subfamily B member 9; FGN, fibrillary glomerulonephritis; HCQ, hydroxychloroquine; IHC, immunohistochemistry; LN, lupus nephritis; NR, not reported; RAASi, renin-angiotensin-aldosterone system inhibitor; SCr, serum creatinine; SLE, systemic lupus erythematosus; UPCR, urine protein-creatinine ratio.

The coexistence of SLE and DNAJB9-positive FGN without LN is exceptionally rare and of uncertain pathophysiologic significance. The Mayo Clinic cohort of 66 patients with FGN documented SLE in only 2 patients (∼3%), both lacking histologic evidence of LN. Similarly, LN biopsies have occasionally demonstrated fibrillary deposits resembling those seen in FGN, suggesting morphologic overlap between these entities.^4^^,^^17^ Both conditions present with overlapping clinical features, including nephrotic-range proteinuria, hematuria, and kidney insufficiency, which can complicate diagnosis. However, DNAJB9 positivity, characteristically absent in LN, remains highly specific for FGN and is a crucial diagnostic tool for distinguishing FGN from morphologic mimics. This overlap in clinical, histologic, and serologic features may suggest a potential etiologic relationship. Despite these observations and scattered case reports of patients with established SLE and biopsy-proven FGN, there is no evidence to date implicating SLE as a causative factor in FGN. In the absence of compelling evidence, we speculate that the concurrent presentation of SLE and FGN may reflect 2 coincidental, independent autoimmune processes or that SLE-mediated immune dysregulation contributes to aberrant immune-complex formation, impaired clearance, or a glomerular microenvironment conducive to fibrillogenesis.

The rarity of this association also precludes an established treatment approach. Of the 3 previously reported cases, only 2 included treatment outcomes, both showing remission with glucocorticoids and mycophenolate. To our knowledge, our report is the first to demonstrate proteinuria remission with rituximab, with creatinine normalization in one patient and preserved function in the other. These findings support B-cell depletion as a rational therapeutic approach in SLE-associated FGN and provide a mechanistic justification for the use of rituximab as a safe and effective alternative in this immune-complex deposition disease. The success of this steroid-sparing regimen is particularly relevant in light of mounting evidence documenting the toxicities of glucocorticoid therapy and a growing consensus within the field to minimize steroid exposure whenever possible.^18^^,^^19^

The natural history of FGN remains poor, with 44%-52% of patients progressing to kidney failure within 4 years.^2^^,^^4^^,^^11^ Although partial remission or disease stabilization may occur with immunosuppression, complete remission is uncommon, and long-term kidney survival remains unfavorable. The natural history of SLE-associated FGN (with or without LN) is also unknown. The only available data come from case reports (included in Table 1).^12^, ^13^, ^14^ Poor prognostic indicators include higher baseline creatinine, nephrotic-range proteinuria, and greater glomerular sclerosis on biopsy.^4^ However, it remains unclear whether treatment response and disease course differ in patients with SLE-associated FGN from isolated FGN. Nonetheless, 4 of the 5 patients described in this report (including our 2 cases) responded to treatment regimens involving steroids, mycophenolate, or rituximab (Table 1).

## Conclusion

FGN remains a rare and challenging glomerular disease with poor long-term kidney survival. An increasing number of SLE-associated FGN cases have been reported; however, the etiologic link remains unclear. In this series, we add 2 cases to the previously published 3 cases of SLE-associated FGN. Although further data are needed to clarify the significance of this association, possible explanations include either the coincidental overlap of independent autoimmune processes or SLE-driven immune dysregulation promoting aberrant immune-complex formation and fibrillogenesis. Equally important, our report provides the first published evidence supporting the safe and effective use of rituximab in this setting as a steroid-sparing alternative.
